# Neuronal Differentiation and Exosome Profiling of Dental Pulp Stem Cells: Unveiling Their Potential for Nerve Repair

**DOI:** 10.3390/ijms26199723

**Published:** 2025-10-06

**Authors:** Bruna Lopes, Patrícia Sousa, Alícia de Sousa Moreira, Ana Catarina Sousa, Alexandra Rêma, Luís Atayde, António J. Salgado, Stefano Geuna, Rui Alvites, Ana Colette Maurício

**Affiliations:** 1 Departamento de Clínicas Veterinárias, Instituto de Ciências Biomédicas de Abel Salazar (ICBAS), Universidade do Porto (UP), Rua de Jorge Viterbo Ferreira, n° 228, 4050-313 Porto, Portugal; brunisabel95@gmail.com (B.L.); pfrfs_10@hotmail.com (P.S.); alicia.moreira.1998@gmail.com (A.d.S.M.) anacatarinasoaressousa@hotmail.com (A.C.S.); alexandra.rema@gmail.com (A.R.); ataydelm@gmail.com (L.A.); ruialvites@hotmail.com (R.A.); 2 Centro de Estudos de Ciência Animal (CECA), Instituto de Ciências, Tecnologias e Agroambiente da Universidade do Porto (ICETA), Rua D. Manuel II, Apartado 55142, 4051-401 Porto, Portugal; 3 Associate Laboratory for Animal and Veterinary Science (AL4AnimalS), 1300-477 Lisboa, Portugal; 4 RISE-Health, School of Medicine and Biomedical Sciences, Fernando Pessoa University, Fernando Pessoa Teaching and Culture Foundation, Avenida Fernando Pessoa 150, 4420-096 Gondomar, Portugal; 5 Biomedical and Health Sciences (FP-BHS), Instituto de Investigação, Inovação e Desenvolvimento Fernando Pessoa (FP-I3ID), Fundação Ensino e Cultura Fernando Pessoa, Praça 9 de Abril, n° 349, 4249-004 Porto, Portugal; 6 Campus Agrário de Vairão, Centro Clínico de Equinos de Vairão (CCEV), Rua da Braziela n° 100, 4485-144 Vairão, Portugal; 7 ICVS/3B’s—PT Government Associated Laboratory, Campus de Gualtar, Universidade do Minho, 4710-057 Braga, Portugal; asalgado@med.uminho.pt; 8 Department of Clinical and Biological Sciences, Cavalieri Ottolenghi Neuroscience Institute, University of Turin, Ospedale San Luigi, 10043 Turin, Italy; stefano.geuna@unito.it; 9 Departamento de Ciência Animal e Veterinária, Instituto Universitário de Ciências da Saúde, Cooperativa de Ensino Superior Politécnico e Universitário (IUCS-CESPU), Avenida Central de Gandra 1317, 4585-116 Gandra, Portugal

**Keywords:** biomaterials, conditioned media, exosomes, human dental pulp stem cells, peripheral nerve injury, regenerative medicine

## Abstract

Peripheral nerve injuries remain a major clinical problem, and cell-free therapies using stem cell-derived bioproducts have emerged as promising alternatives. This study evaluated the influence of neurogenic differentiation and passage number on the secretomic and exosomal profile of human dental pulp stem cells (hDPCSs). Conditioned media from undifferentiated and neurodifferentiated hDPSCs, and exosomes derived from undifferentiated hDPSCs at passages 4 and 7, were analyzed using multiplex immunoassays, RT-PCR, and scanning electron microscopy (SEM). Neurodifferentiated hDPSCs at early passages secreted higher levels of neurotrophic, angiogenic and immunomodulatory factors, including FGF-2, IL-6, IL-8, and PDGF-AA. Exosomes from early-passage undifferentiated cells showed a more abundant and relevant neuroregenerative mRNA cargo in comparison to the later passages. Both cell types and exosomes adhered to the Reaxon^®^ nerve guidance conduit, confirming the permissive nature of the materials regarding cells and cellular products, allowing adhesion and survival. Neurite outgrowth assays performed on neurodifferentiated hDPSCs confirmed functional neural behavior. In later passages, a decline in secretory and exosomal activity was noted. These results highlight the relevance of early-passage hDPSCs as a source of bioactive factors and support their application in cell-free approaches for peripheral nerve regeneration.

## 1. Introduction

Peripheral nerve injuries (PNIs) are a prevalent cause of sensory and motor deficits, often resulting from trauma, surgical procedures, or diseases [1]. Defects smaller than 4 cm usually have better outcomes, with regeneration occurring at a slow rate of 1–3 mm per day, varying with the injury’s complexity and location [2,3]. Despite the peripheral nervous system’s intrinsic regenerative capacity, the functional recovery following extensive nerve damage remains suboptimal [4]. So, defects larger than 4 cm result in worse outcomes due to a lack of effective regeneration [2]. In these situations, neurorrhaphy and autologous nerve grafting remain the current gold standard techniques, yet they present significant drawbacks such as donor site morbidity, limited availability, and inconsistent outcomes [3]. Besides these difficulties, the peripheral nerve regeneration depends on the injury’s type and severity [5]. In response to these limitations, regenerative medicine has emerged as a promising field, with cell-based and, more recently, cell-free therapies gaining considerable interest for their ability to modulate the microenvironment and promote nerve repair [2,6].

To address the limitations of autologous grafting, several strategies have been developed to enhance peripheral nerve regeneration. Various studies developed techniques for preserving functional nerves by promoting axonal regeneration and enhancing recovery from PNI [7,8,9]. Nerve Guide Conduits (NGCs) are a viable option for nerve repair, used to bridge the severed ends of the lesioned nerve, without sacrificing any healthy nerve for transplantation [1,10]. These devices provide a protected environment that supports and guides the axonal growth, cell migration, and extracellular matrix deposition, contributing to functional recovery [8]. However, in long-gap injuries, NGCs alone show limited effectiveness [11,12]. For that reason, the combination of NGC with stem cells and their secretions is one of the possibilities for an ideal treatment [2,9].

In peripheral nerve regeneration, mesenchymal stem cells (MSCs) have an important role due to their ability to produce and release a diverse collection of molecular and biochemical factors, denominated as secretome or its counterpart after conditioning, the conditioned medium (CM) [13]. Among the various cellular sources investigated, human dental pulp stem cells (hDPSCs) have drawn particular attention due to their neurotropic characteristics, ease of isolation, high proliferation, self-renewal capacity, multilineage differentiation and low immunogenicity [10,14,15]. Their capacity to differentiate toward neuronal and glial lineages, along with the secretion of bioactive molecules with trophic, angiogenic, and immunomodulatory properties, justifies their use in neuroregenerative therapies [16,17], and their self-renewing ability and pronounced plasticity support their application across various medical contexts [18]. However, despite their potential, the clinical implementation of stem cell-based therapies remains limited due to technical and biological limitations [19,20]. One of the main concerns is the safety, particularly the risks associated with immune rejection, uncontrolled cell proliferation, and pro-inflammatory responses [19,21,22], which, although rare, considering the immunoprivileged nature of these cells, can still occur. An alternative mechanism of therapeutic action has been proposed, highlighting the paracrine role of MSCs, the CM from hDPSCs (hDPSCs-CM) and hDPSC-derived exosomes [1]. Specifically, hDPSCs-CM has emerged as a cell-free therapeutic agent that contains a rich mixture of soluble factors capable of modulating inflammation, promoting angiogenesis, and enhancing neural regeneration [23,24]. Building on this concept, research has supported the beneficial effects of MSCs predominantly mediated by the molecules they secrete [25,26]. Valadi et al. were among the first to describe the transfer of mRNA between cells mediated by extracellular vesicles (EVs), suggesting that genetic communication could occur independently of direct cell integration [27]. EVs, particularly exosomes, stand out due to their unique biogenesis and complex molecular cargo, positioning them as key mediators of MSC-induced tissue regeneration [28,29]. For that reason, hDPSCs-CM and hDPSC-derived exosomes have been increasingly recognized as critical effectors of tissue repair, acting via paracrine mechanisms rather than direct cell replacement [30,31,32].

According to the guidelines established by the International Society for Extracellular Vesicles (ISEV), the characterization of EVs must fulfill three essential criteria: (1) EVs must be isolated from conditioned cell culture media or biological fluids, ensuring minimal cellular disruption during the process; (2) at least one protein from each of three distinct categories should be identified in the EV preparation: cytosolic proteins, transmembrane or lipid-bound extracellular proteins, and intracellular proteins; (3) the vesicles must be characterized using at least two complementary techniques—one based on imaging, such as electron microscopy, and another that determines size distribution [33,34]. Exosomes are nanometric vesicles (30–150 nm) that encapsulate proteins, lipids, and nucleic acids, notably mRNA and microRNA, playing a crucial role in intercellular communication [35,36]. When derived from stem cells, exosomes carry neurotrophic and anti-inflammatory signals, influencing processes such as axonal outgrowth, Schwann cell activation, immune modulation, and facilitating cell proliferation and enhancing angiogenesis and regeneration [35,37,38]. Recent studies have highlighted the potential of hDPSC-derived exosomes to contribute to peripheral nerve regeneration [39,40,41].

The present study aims to investigate the influence of neurogenic differentiation and cellular passage number on the secretory and vesicular profiles of hDPSCs, with relevance to their application in peripheral nerve regeneration. To this end, the viability and adhesion of neurodifferentiated hDPSCs and exosomes derived from hDPSCs, cultured within a commercially available NGC (Reaxon^®^), were evaluated to determine their compatibility with the biomaterial. Simultaneously, the paracrine activity of hDPSCs was assessed through quantification of cytokines and growth factors in the CM, alongside transcriptomic characterization of exosomes derived from undifferentiated cells at P4 and P7. A comprehensive methodological approach, comprising multiplex immunoassays, reverse transcription PCR, total protein content, and scanning electron microscopy, was employed to characterize the functional CM and exosomes. In addition, a neurite outgrowth assay was performed in different conditions to assess hDPSC behavior. These findings are expected to contribute to the development of optimized cell-free therapeutic approaches and to support the incorporation of hDPSC-derived exosomes and hDPSCs-CM in future in vivo models for peripheral nerve repair.

## 2. Results

### 2.1. Analysis of hDPSC Conditioned Medium and hDPSC Exosomes

**Figure 1:**
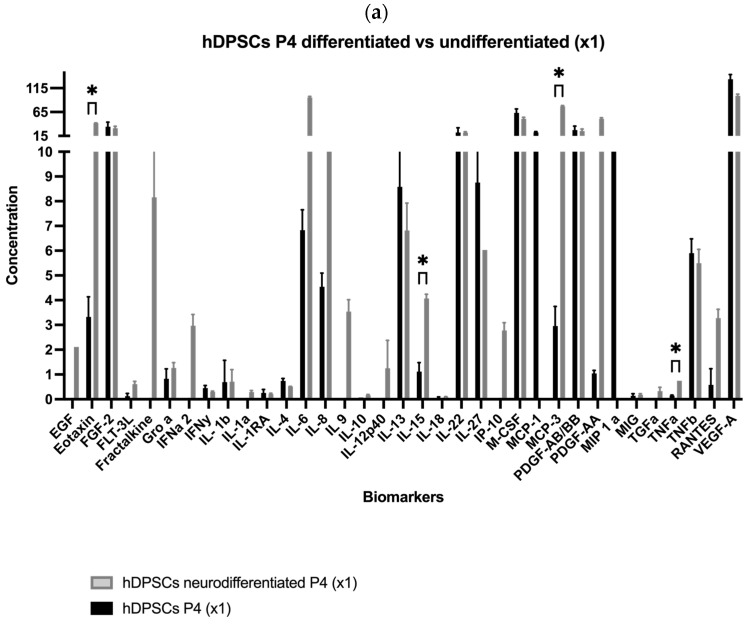

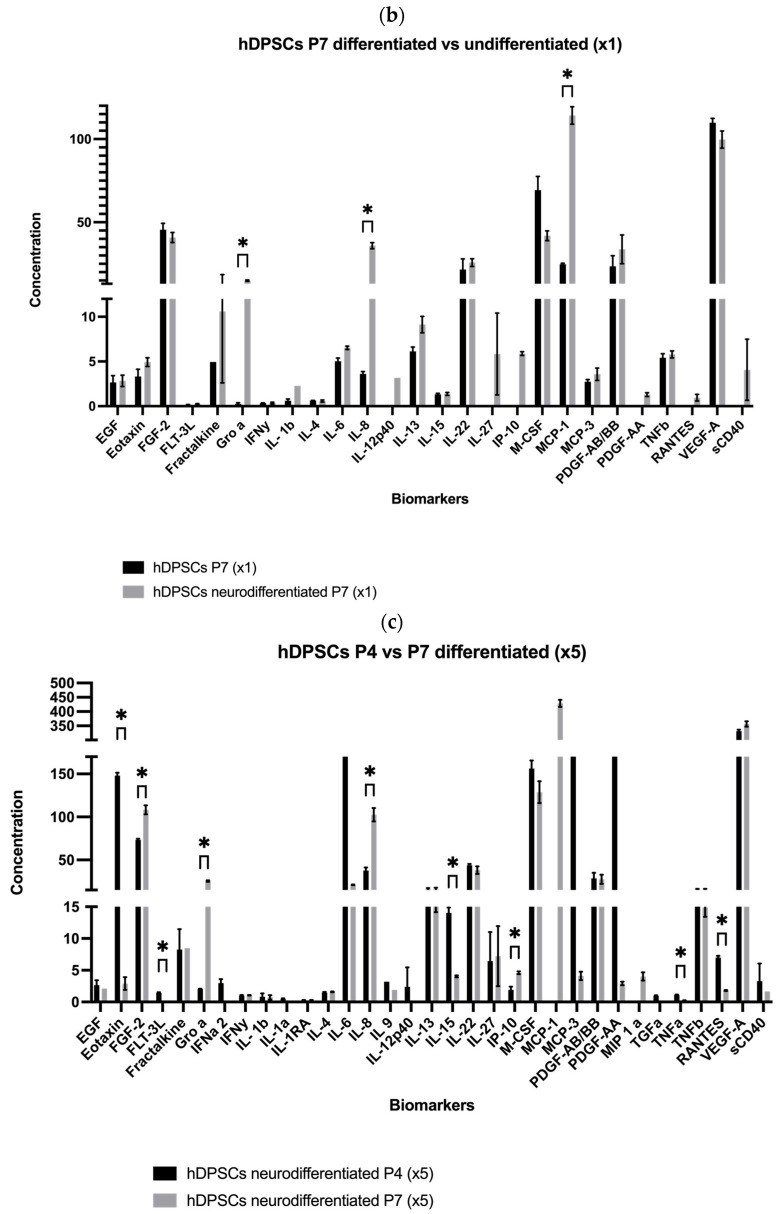

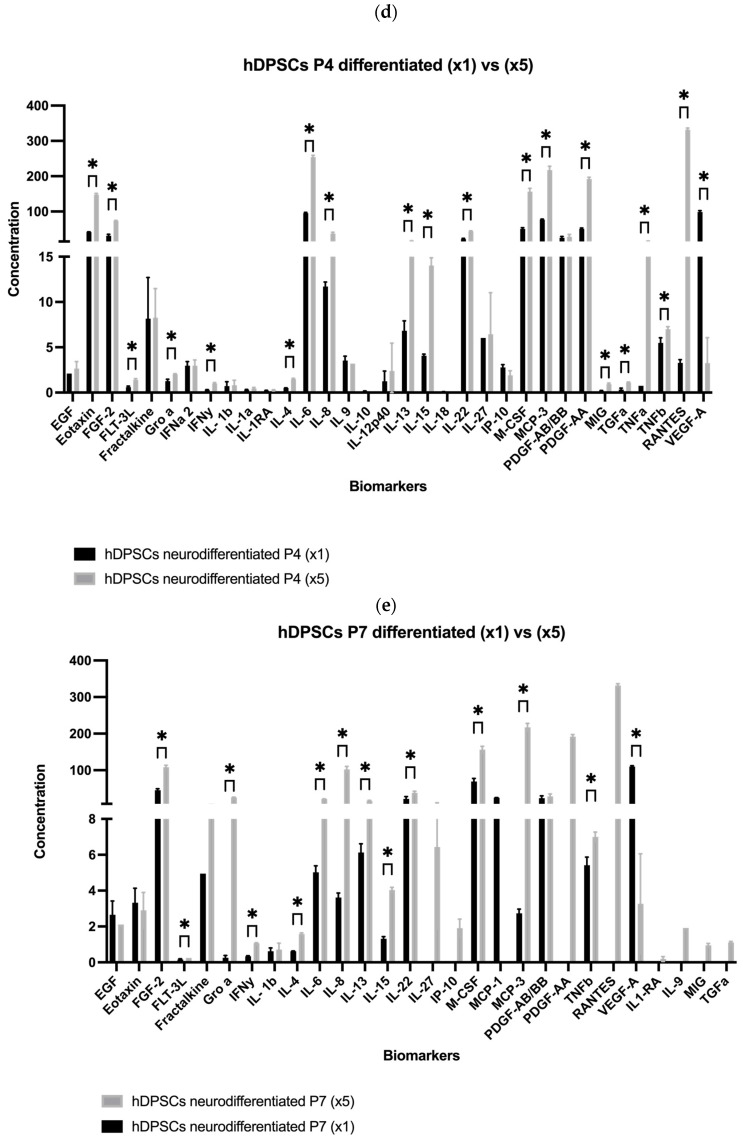

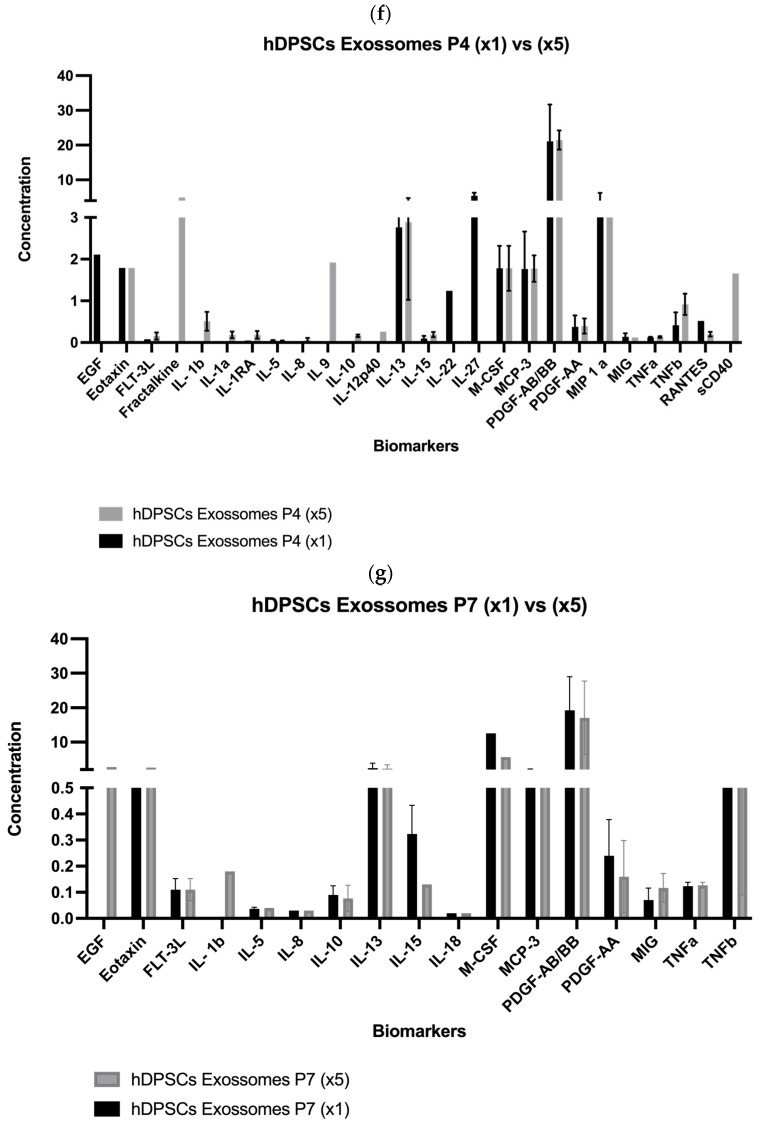
(**a**–**e**) Concentration of each biomolecule (pg/mL) present in the CM-hDPSCs under different experimental conditions (mean ± SEM). (**f**,**g**) Concentration of each biomolecule (pg/mL) present in exosomes derived from hDPSCs (mean ± SEM). The significance of the results is indicated by symbols (*), with (*) corresponding to 0.01 ≤ *p* < 0.05.

Results for the hDPSC conditioned medium are presented in Table 1 and Figure 1a–e, and results for the hDPSC exosomes are presented in Table 2 and Figure 1f,g. To evaluate the impact of neurogenic differentiation and passage number on the secretory activity of hDPSCs, both CM and exosomes were analyzed under various experimental conditions. In the CM fraction, comparisons were made between undifferentiated and neur-differentiated hDPSCs at P4 and P7, under both 1× and 5× concentrations.

At 1× concentration, neurodifferentiated hDPSCs at passage 4 secreted higher levels of EGF, FLT-3L, Fractalkine, GRO-α, IFNα2, IL-1α, IL-6, IL-8, IL-9, IL-10, IL-12p40, IP-10, PDGF-AA, and RANTES, compared to CM from undifferentiated cells. Among these, Eotaxin, IL-15, MCP-3, and TNF-α showed statistically significant increases, indicating an enhanced neurotrophic, angiogenic, and immunomodulatory profile [2,42]. While most analytes were detected under both conditions, certain molecules were found exclusively in one group. For instance, EGF, IL-9, and IL-10 were detected only in the neurodifferentiated condition, whereas MIP and MCP-1 were exclusively present in the undifferentiated group. This selective expression pattern suggests that neuronal differentiation modulates specific components of the CM rather than inducing a uniform up- or downregulation. These findings highlight that early passage neurodifferentiated hDPSCs exhibit a robust secretory profile enriched in pro-inflammatory, immunoregulatory, and neuroactive factors [26].

At passage 7, still under 1× concentration, neurodifferentiated hDPSCs showed moderately increased levels of several cytokines, including EGF, Eotaxin, Fractalkine, IL-1β, IL-6, IL-12p40, IL-13, IL-22, IL-27, IP-10, MCP-3, PDGF AB/BB, PDGF-AA, RANTES, and sCD40, compared to undifferentiated cells. Conversely, FGF-2, M-CSF, TNF-β, and VEGF-A were higher in the undifferentiated condition. Statistically significant increases were observed for MCP-1, IL-8, and GRO-α in the neurodifferentiated group. Although the general pattern of change was like the one observed at P4, the magnitude of these differences was attenuated.

As expected, increasing the concentration from 1× to 5× led to a proportional rise in cytokine detection across all conditions. At P4, concentration enhanced levels of Eotaxin, FGF-2, IL-6, IL-22, M-CSF, MCP-3, PDGF-AA, and VEGF-A. In P7 samples, notable increases were observed in FGF-2, IL-8, M-CSF, MCP-1, and RANTES. These findings indicate that while concentration improves detection sensitivity, the intrinsic secretory output remains influenced by replicative senescence [43].

To further clarify the effect of passage number on neurodifferentiated hDPSCs, a direct comparison between P4 and P7 was conducted using 5× concentrated CM. The analysis revealed a general decrease at P7 in the secretion of EGF, Eotaxin, FLT-3L, IFNα2, PDGF-AA, RANTES, M-CSF, MCP-3, IL-6, IL-9, IL-12p40, IL-15, sCD40, and TNF-α. Notably, PDGF-AA plays a key role in mitogenic stimulation and tissue repair, while the decline in TNF-α may reflect reduced immunomodulatory activity [44]. Nevertheless, some factors, such as VEGF-A and PDGF-AB/BB, remained relatively stable between passages, indicating that not all components of the CM are equally affected by senescence. Interestingly, MCP-2 was detected only at P7, and both GRO-α and FGF-2 exhibited increased levels in this later passage, further emphasizing the selective nature of the observed secretory changes, as seen in Table 1.

Exosomes derived from undifferentiated hDPSCs were also evaluated at P4 and P7 under both 1× and 5× concentrations. Both concentration and passage number influenced the exosomal profile. At P4, exosome preparations displayed elevated levels of cytokines and growth factors, including Fractalkine, IL-1α, IL-22, IL-27, MCP-3, PDGF-AB/BB, and MIP-1α, all of which are implicated in immune modulation, neuroprotection, and regenerative signaling [45]. In contrast, P7-derived exosomes exhibited a generalized reduction in these molecules, particularly PDGF-AB/BB and MIP-1α, even after concentration. This suggests a passage-dependent decline in exosome-mediated signaling capacity. Although some analytes, such as EGF, Eotaxin, IL-5, IL-10, IL-8, and TNF-α, remained relatively stable across passages, most exosome-associated factors showed decreased abundance with advancing passage, as detailed in Table 2.

In summary, the secretome and exosomal profiles of hDPSCs are dynamically regulated by both differentiation state and passage number. Neurodifferentiation, particularly at early passages, enhanced the secretion of neurotrophic and immunomodulatory factors in the CM. The summary of the effects of neurodifferentiation, passage number, and concentration on key biomarkers in hDPSC-CM and their main biological roles is presented in Table 3. Furthermore, even exosomes derived from undifferentiated hDPSCs retained higher concentrations of key regenerative signals at early passages.

### 2.2. Scanning Electron Microscopy (SEM) and Energy Dispersive X-Ray Spectroscopy (EDS) Analysis

**Figure 2:**
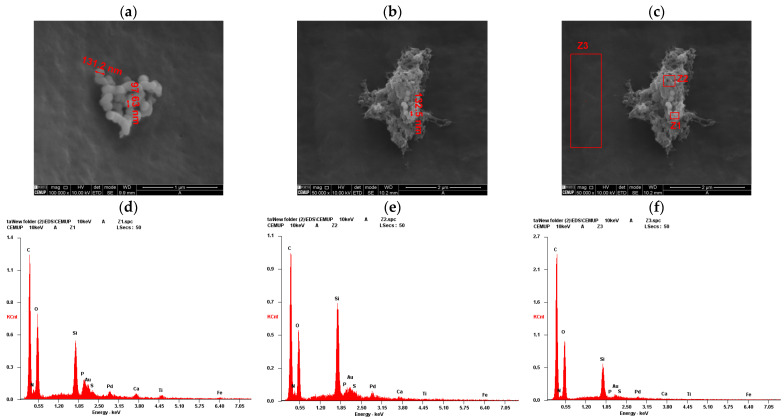
SEM with EDS analysis of hDPSC-Derived Exosomes: (**a**,**b**) SEM image of exosomes with measurement of exosome dimensions; (**c**) identification of exosome regions for elemental analysis; (**d**) EDS spectrum of exosome region Z1; (**e**) EDS spectrum of exosome region Z2 and (**f**) EDS spectrum of exosome region Z3.

**Figure 3:**
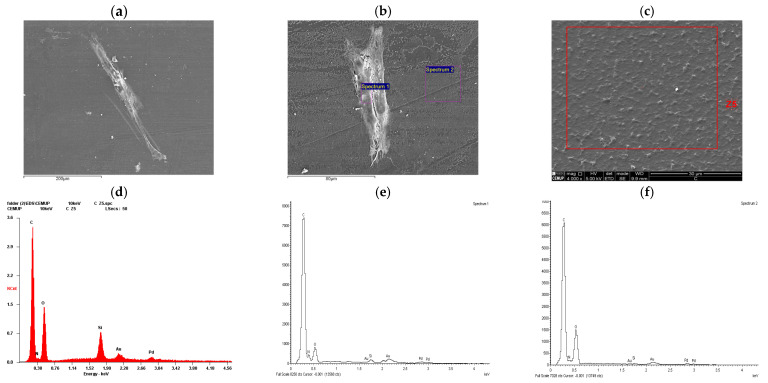
SEM and EDS evaluation of the Reaxon^®^ NGC and hDPSCs neurodifferentiated: (**a**) hDPSC cell layer adhered to the inner face of the Reaxon^®^ NGC, magnification: 2000×; (**b**) identification of hDPSC regions for elemental analysis; (**c**) inner surface of the Reaxon^®^ tube without cells; (**d**) EDS evaluation of Z5 region; (**e**) EDS evaluation of the Spectrum 1 region; (**f**) EDS evaluation of the Spectrum 2 region.

The outcomes of the scanning electron microscopy and energy dispersive X-ray Spectroscopy from hDPSCs can be found in Figure 2, and from hDPSC exosomes in Figure 3. Following the evaluation of hDPSCs-CM and hDPSC-derived exosomes, samples were prepared for scanning electron microscopy (SEM) and energy dispersive X-ray spectroscopy (EDS) analysis. This analysis was performed to assess the interaction between the tested biomaterial Reaxon^®^ (Medovent GmbH (Mainz, Germany)) and both neurodifferentiated hDPSCs and hDPSC-derived exosomes.

SEM imaging of hDPSC-derived exosomes (Figure 2a,b) revealed uniformly distributed vesicles with spherical to slightly concave morphology and diameters predominantly ranging from 100 to 130 nm, consistent with typical exosome characteristics [46,47]. Their homogeneous appearance and preserved membrane integrity suggest successful isolation and structural stability. EDS analysis confirmed the biological nature of these vesicles. In regions containing visible exosomes (Z1 and Z2; Figure 2c), elements such as carbon, nitrogen, oxygen, phosphorus, and sulfur were detected, consistent with lipid membranes and nucleoprotein content (Figure 2d,e). In contrast, the control region without vesicles (Z3; Figure 2c) showed an elemental profile dominated by silicon, calcium, and titanium, attributed to the scaffold or residual medium (Figure 2f). Gold and palladium, used for sputter coating, were present in all spectra. These findings confirm the exosomal identity and demonstrate their ability to adhere to the biomaterial surface.

Similarly, SEM images of neurodifferentiated hDPSCs cultured on the inner surface of the Reaxon^®^ conduit (Figure 3a,b) revealed scattered adherent cells exhibiting elongated morphology and cytoplasmic projections, characteristic of early neural-like differentiation, but a continuous monolayer was not observed. EDS results from the cell-covered region (Spectrum 1; Figure 3b) revealed a biological elemental signature, including carbon, nitrogen, and oxygen, confirming the presence of cellular material (Figure 3e). In contrast, results from an uncovered region (Spectrum 2; Figure 3b) lacked nitrogen and other organic markers, further supporting the specificity of the signals in the cell-adherent area (Figure 3f). SEM imaging of the inner surface of the Reaxon^®^ conduit without cells (Figure 3c) shows a uniform microstructured pattern characteristic of the biomaterial’s architecture. Elemental analysis by EDS of the Z5 region (Figure 3d) shows carbon and oxygen as the predominant elements, consistent with the polymeric composition of the conduit, with traces of silicon, gold, and palladium from the sputter-coating process. This profile matches the expected composition of the conduit surface before cell seeding.

Together, these observations confirm that the Reaxon^®^ scaffold supports both the adhesion and morphological adaptation of neurodifferentiated hDPSCs, as well as the stable interaction of exosomes with its surface.

### 2.3. Reverse Transcriptase Polymerase Chain Reaction (RT-PCR)

**Figure 4:**
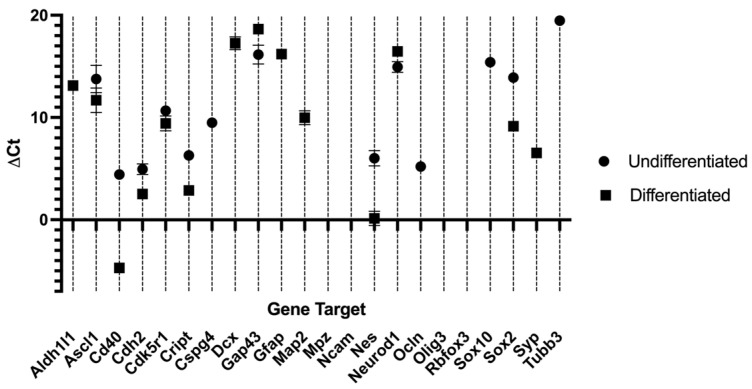
∆Ct values for the different genes under study in hDPSCs, undifferentiated and after neurogenic differentiation. Higher ∆Ct values represent lower expression. Results are presented as mean ± SEM.

**Figure 5:**
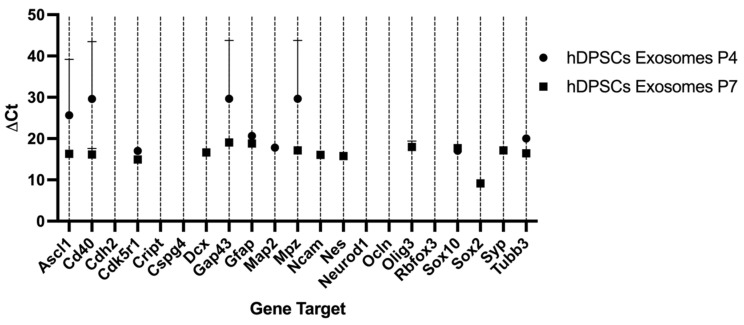
∆Ct values for the different genes under study in hDPSC exosomes in the different passages. Higher ∆Ct values represent lower expression. Results are presented as mean ± SEM.

The outcomes of the RT-PCR of hDPSCs can be found in Table 4 and Figure 4, and from hDPSC exosomes in Table 5 and Figure 5. Mean Ct values for each target gene, along with the corresponding ΔCt, ΔΔCt, and RQ calculations, are presented in Table 4. RNA purity, assessed by UV spectrophotometry, confirmed that all samples met the required quality standards, ensuring their suitability for downstream analyses. The melting curve profiles displayed a single peak for each gene, indicating the specific amplification of a single amplicon. The transcriptional profile of hDPSCs was evaluated by RT-PCR to assess the impact of neurogenic differentiation. Several genes associated with early neurogenesis and neural progenitor identity, including *Ascl1*, *Nes*, *Cdk5r1*, *Sox2*, *Cd40*, *Cdh2* and *Cript*, were significantly upregulated in neurodifferentiated cells, suggesting activation of neurodevelopmental pathways [8]. Similarly, markers of neuronal maturation, *Map2*, *Dcx*, *Syp*, and astroglial lineage commitment, *Gfap*, *Aldh1l1*, were strongly increased following differentiation, further supporting lineage specification toward neural phenotypes. Interestingly, *Tubb3*, commonly used as an early neuronal marker, was detected only in undifferentiated cells and absent in differentiated ones [48]. This pattern may reflect basal expression in the progenitor state and a transient downregulation during the induction process, or alternatively, an insufficient activation under the specific conditions applied. Some genes, such as *Gap43*, showed a downregulation between groups, suggesting they are not strongly modulated during early differentiation. Others, such as *Mpz*, *Ncam1*, *Rbfox3* and *Olig3*, were undetected in both groups, possibly due to low expression levels or non-relevance to the specific neuroglial pathways engaged in this model. For transcripts not identified in either undifferentiated or neurodifferentiated cells, relative expression could not be determined.

Together, these results demonstrate that neurogenic induction in hDPSCs elicits a transcriptional shift characterized by increased expression of neuroprogenitor, neuronal, and astroglial markers, while certain lineage markers remain unaffected or undetectable.

The analysis of 22 neuroglial-related genes in exosomes derived from hDPSCs revealed a selective expression pattern and significant variation between P4 and P7. Overall, a greater number of transcripts were detected in P7 exosomes, although many exhibited low expression levels. Notably, *Cd40* and *Cdk5r1* were significantly upregulated in P7 compared to P4. In contrast, several neuroglial markers, including *Tubb3*, *Gfap*, and *Mpz*, showed reduced expression in P7-derived exosomes. *Tubb3* was strongly downregulated, suggesting a diminished neurogenic content in vesicles from late-passage cells. *Gfap* and *Mpz* also declined modestly. Other genes such as *Ascl1*, *Sox10*, and *Gap43* showed minimal changes between passages, indicating stable expression across conditions.

Certain transcripts were exclusively detected in one passage. *Dcx*, *Ncam*, *Nes*, *Olig3*, *Syp*, and *Sox2* were only present in P7-derived exosomes, while *Aldh1l1*, *Cdh2*, *Cript*, *Cspg4*, *Ocln*, *Rbfox3*, and *Neurod1* were undetectable under both conditions. This observation suggests that specific gene loading into exosomes is influenced by passage-dependent regulatory mechanisms and may not mirror cellular transcript abundance, as observed in Table 5.

Taken together, these findings demonstrate that the exosomal mRNA cargo is selectively modulated by passage number.

### 2.4. Neurite Outgrowth Assay

**Figure 6:**
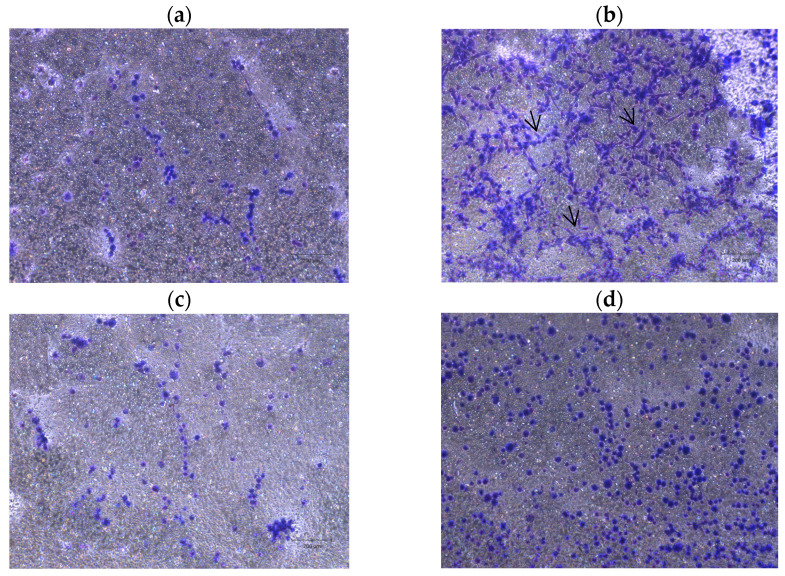
Representative images of neurite outgrowth in hDPSCs under different conditions: (**a**) BSA coated inserts (2 mg/mL), serum free media (negative control), (**b**) laminin coated inserts (10 µg/mL), serum free media, (**c**) laminin coated inserts, serum free media plus nocodazole 250 ng/mL 0 h (from the beginning of differentiation), and (**d**) laminin coated inserts, serum free media plus nocodazole 250 ng/mL 24 h (Cells differentiated for 24 h, followed by the addition of 250 ng/mL nocodazole for another 24 h). Neuritic projections (black arrow) on the basal membrane surface reflect differential modulation of neurite extension. Scale bar: 200 µm.

**Figure 7:**
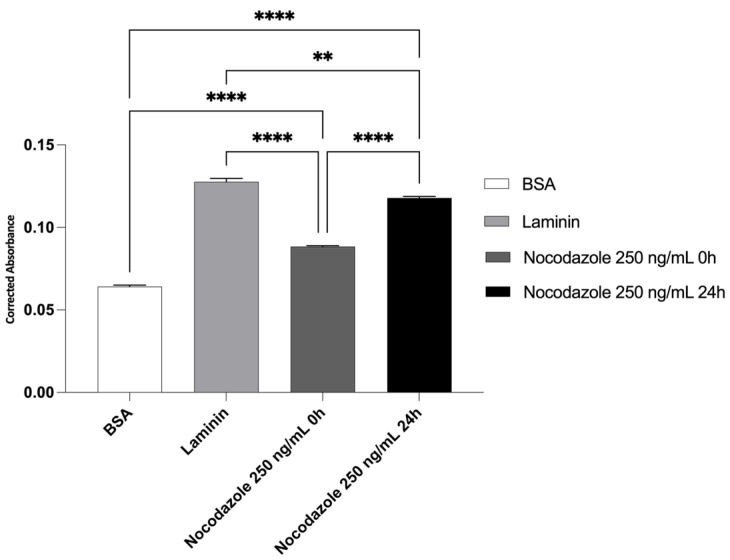
Quantification of neurite outgrowth in hDPSCs under different conditions. Conditions included BSA (negative control), laminin (positive control), and nocodazole treatment applied at 0 h or 24 h of differentiation. Data are expressed as mean ± SEM. Results significances are presented through the symbol (*), according to the *p*-value, two or four symbols, corresponding to 0.001 < *p* ≤ 0.01 and *p* ≤ 0.0001.

The outcomes of the neurite outgrowth assay for hDPSCs are shown in Figure 6, and the quantitative analysis is presented in Figure 7. Neurite outgrowth was assessed in differentiated hDPSCs cultured on 1 µm pore Millicell inserts. After a 48 h incubation period, neuritic projections crossing the membrane were visualized by staining the basal membrane surface.

In the condition where membranes were coated with BSA (Figure 6a), the basal side showed only faint and sparse staining, with no evident linear or filamentous structures. The signal was mostly diffuse and punctate, indicating an absence of organized neuritic projections. This minimal staining is consistent with negligible neurite extension and supports the use of BSA as a negative control substrate.

In contrast, membranes coated with laminin (Figure 6b) exhibited intense, well-defined staining on the basal surface. Many elongated, fiber-like structures were visible, displaying a radiating pattern indicative of directional neurite extension through the pores. The distribution of the staining was widespread and homogeneous across the membrane surface, reflecting neuritic outgrowth.

When nocodazole (250 ng/mL) was added from the beginning of differentiation (Nocodazole 0 h—Figure 6c), neurite staining was markedly reduced. Only sparse and discontinuous projections were detected on the basal surface, suggesting early and sustained disruption of microtubule dynamics that inhibited neurite formation.

In the condition where nocodazole was introduced after 24 h of differentiation (Nocodazole 24 h—Figure 6d), neurite staining was reduced relative to the standard differentiation condition but more pronounced than in the Nocodazole 0 h group. Some less marked neuritic extensions remained visible, indicating that pre-established neurites may be partially resistant to delayed nocodazole exposure.

Quantification of neurite outgrowth was performed by extracting the membrane-bound stain and measuring absorbance at 570 nm. The quantitative analysis of neurite outgrowth (Figure 7) showed significantly increased absorbance in the laminin group, confirming its neuroinductive potential. BSA-coated membranes presented minimal absorbance, validating the absence of neurite formation. Nocodazole treatment from 0 h reduced neurite-associated absorbance, and when applied at 24 h, absorbance remained high, suggesting partial resistance of pre-established neurites to depolymerizing conditions. To specifically quantify the number of cells, the QuPath v0.6.0 application was used. The analysis showed 733 cells in the BSA–coated wells, 2728 cells in the laminin-coated wells, 474 cells in the wells treated with nocodazole from the beginning of differentiation, and 1257 cells in the wells treated with nocodazole after 24 h of differentiation.

### 2.5. Total Protein Quantification

**Figure 8:**
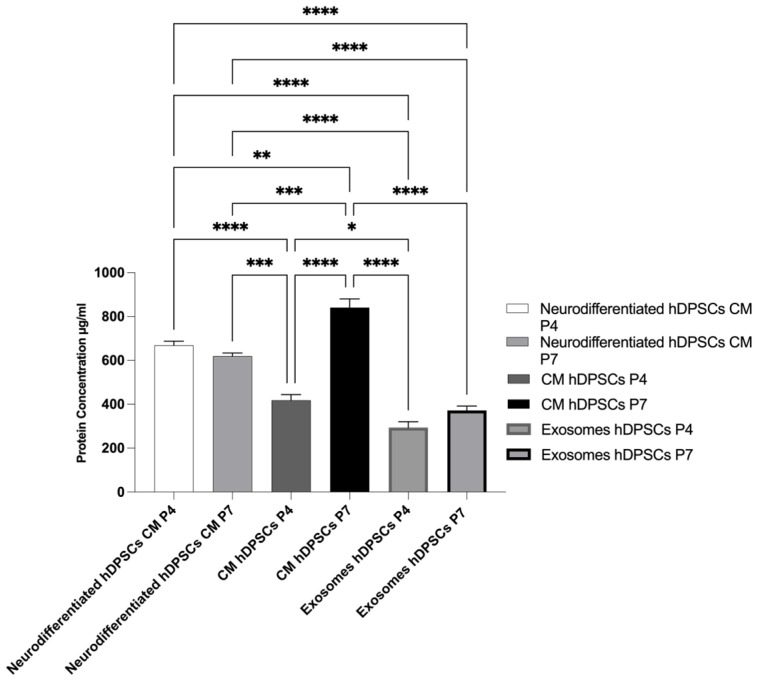
Protein concentration for secretome and exosomes isolated from cells in P4 and P7 (mean ± SEM). Results significances are presented through the symbol (*), according to the *p*-value, with one, two, three, or four symbols, corresponding to 0.01 < *p* ≤ 0.05; 0.001 < *p* ≤ 0.01; 0.0001 < *p* ≤ 0.001 and *p* ≤ 0.0001.

The outcomes of the total protein quantification of different groups can be found in Figure 8, and the statistical differences are present in Table 6. The secretory activity of hDPSCs was influenced by both neurodifferentiation and cell passage number. This modulation was reflected in the composition of the CM, the cytokine profiles associated with exosomes, and the total protein content.

Total protein concentration was quantified in the CM of both undifferentiated and neurodifferentiated hDPSCs, as well as in hDPSC-derived exosomes, at P4 and P7. As shown in Figure 8, CM samples exhibited substantially higher protein concentrations than the corresponding exosomal fractions, reflecting the broader composition of the CM, which includes soluble proteins, cytokines, and growth factors in addition to vesicle-associated components. In contrast, the exosomal fraction represents a more selective subset, enriched in vesicle-specific cargo.

In neurodifferentiated cells, total protein levels were slightly higher at P4 compared to P7, indicating that neurodifferentiation enhances the secretory activity, particularly in early passages. Interestingly, in undifferentiated cells, the opposite pattern was observed: both CM and exosome fractions showed higher total protein content at P7 than at P4. Notably, the highest protein concentration was detected in the CM from undifferentiated P7 cells. This unexpected increase suggests a possible shift in basal secretory activity or altered metabolic behavior in late-passage undifferentiated cells.

Altogether, these results show that neurodifferentiation promotes increased protein secretion in early passages, while undifferentiated cells at later passages may retain or even enhance protein output.

## 3. Discussion

This study investigated how neurogenic differentiation, passage number, and CM concentration influence the secretory and vesicular profile of hDPSCs by analyzing the composition of the CM and exosomes, as well as their interaction with an NGC. These findings provide relevant insights for optimizing hDPSC-derived products for peripheral nerve regeneration, demonstrating that both neurogenic differentiation and passage number critically modulate the paracrine profiles of hDPSCs, with direct implications for their application in peripheral nerve regeneration. Studies on MSC-CM and exosomes from bone marrow, adipose tissue, and umbilical cord sources have frequently shown enrichment in angiogenic and immunomodulatory cytokines. hDPSC-derived products share these properties but also display distinctive features [49]. Notably, hDPSCs release higher levels of neurotrophic and neuroprotective factors than other MSCs, supporting their relevance for neural repair [50,51]. Evidence also suggests that hDPSC-derived exosomes may promote neurite extension and Schwann cell migration [52,53]. Our findings of enriched VEGF-A, FGF-2, IL-6, and IL-22 in early-passage CM are consistent with Sultan et al. [50,51], who reported that hDPSC-CM supports neuronal survival and differentiation. Likewise, the enhanced neurotrophic and immunomodulatory cargo observed in exosomes parallels previous reports by Pisciotta et al. [54], where hDPSC-derived vesicles promoted peripheral nerve regeneration. Nevertheless, differences between studies in cell passage, isolation protocols, and analytical methods make direct comparisons difficult and highlight the need for standardization [33,45]. Overall, these observations indicate that hDPSCs could serve as a valuable source of regenerative CM and exosomes for peripheral nerve repair, although further standardized studies are needed to fully establish their efficacy [14,30,55]. In addition, direct comparisons with other MSC sources have shown that bone marrow-derived MSCs (BM-MSCs) and adipose-derived MSCs (AD-MSCs) also secrete trophic factors that support nerve regeneration; however, their accessibility and proliferation rates are lower than those of hDPSCs [49,56]. Umbilical cord MSCs (UC-MSCs) possess immunomodulatory and angiogenic properties but appear to have a less pronounced neurotropic profile compared to hDPSCs, which is likely related to the neural crest origin of the latter [54]. This developmental origin likely underlies the greater tendency of hDPSCs to express neural markers and to secrete neuroactive molecules [57]. Together, these features emphasize the translational advantages of hDPSCs, which combine ease of isolation from discarded dental tissue, strong proliferative capacity, and a neuroregenerative secretome, positioning them as a particularly promising source for cell-free nerve repair strategies [2].

In this study, this advantage was particularly evident at early passage (P4): neurodifferentiated hDPSCs exhibited a markedly enhanced secretory profile, particularly in the CM, with increased levels of bioactive molecules, including FGF-2, VEGF-A, PDGF-AA, IL-6, IL-8, IL-22, M-CSF, MCP-3, and Eotaxin. These factors collectively support key regenerative processes such as angiogenesis, neuronal survival, Schwann cell activation, and immune modulation, essential functions for effective repair of injured peripheral nerves [19,49]. Several of these molecules act synergistically [58]. Angiogenic factors like VEGF-A and FGF-2 promote neovascularization and provide trophic support to regenerating tissues [59]. Cytokines such as IL-6 and IL-8 are involved in both inflammation regulation and tissue remodeling [60], while chemokines like MCP-3 and M-CSF enhance macrophage recruitment and polarization, contributing to Wallerian degeneration and the clearance of inhibitory debris [61]. The presence of IL-22 and Eotaxin further points to an activated, repair-oriented immune environment [62]. The coordinated upregulation of these molecules in early-passage, neurodifferentiated cells highlights their regenerative potential and supports their use in cell-free therapeutic strategies [63].

At P7, despite a general attenuation in secretory activity, certain factors remained elevated, namely FGF-2, IL-8, IL-22, M-CSF, MCP-1, and RANTES. This pattern suggests a selective adaptation of the secretory phenotype with cellular aging. Of particular interest is the significant upregulation of RANTES, a chemokine involved in immune cell recruitment, but also increasingly recognized for roles in axonal regeneration, neuroimmune signaling, and remyelination [64]. Its elevation at P7 may reflect a compensatory response to reduced overall secretory capacity, or the onset of a senescence-associated secretory phenotype, where certain inflammatory and chemotactic factors are selectively maintained or amplified [64]. The rise in MCP-1 at P7 may further support this interpretation, as it plays a central role in monocyte/macrophage recruitment and has been implicated in both neuroinflammation and repair [23].

The comparison between 1× and 5× CM concentrations confirmed that concentration amplifies the detection of already abundant cytokines but does not overcome the intrinsic decline in secretion associated with cellular senescence [65]. This suggests that while CM concentration enhances analytical sensitivity, it does not modify the underlying secretory potential, further reinforcing the importance of using early-passage cells.

In parallel, the analysis of exosomes revealed results closely aligned with those observed in the CM. Exosomes derived from P4 hDPSCs contained higher levels of regenerative and immunomodulatory molecules such as Fractalkine, IL-1α, IL-22, IL-27, MCP-3, PDGF-AB/BB, and MIP-1α. These vesicle-associated factors contribute to immune modulation, axonal growth, and glial support, and their abundance in early-passage preparations suggests that exosome-mediated signaling is particularly effective at this stage [66]. In contrast, exosomes derived from P7 hDPSCs exhibited a broad reduction in most of these molecules, even after concentration, indicating a diminished vesicular bioactivity associated with replicative aging [67]. Despite some stability in factors like EGF, IL-8, IL-10, and TNF-α across both passages, the overall decline in exosome content at P7 supports the notion that passage number negatively affects not only the CM but also vesicle-based communication. Interestingly, some factors, such as VEGF-A and PDGF-AB/BB, remained relatively stable, suggesting that a subset of regenerative signals may resist senescence-induced downregulation [68].

Taken together, these findings highlight the dynamic nature of hDPSC paracrine signaling and emphasize that neurodifferentiation promotes an overall increase in the secretion of bioactive proteins and cytokines in the CM, especially in early passages [2]. However, with increasing passage number, a selective and partial preservation of secretory activity is observed. While this may reflect compensatory mechanisms, the global decline in trophic and immunomodulatory content reinforces the importance of controlling passage number in the standardization of hDPSC-derived therapeutic products for use in PNIs therapeutics.

The SEM analysis of neurodifferentiated hDPSCs seeded on the inner surface of the Reaxon^®^ scaffold revealed clear evidence of cell adhesion and morphological adaptation to the substrate. Although the cells did not form a continuous layer, their elongated shapes and cytoplasmic projections suggest effective adhesion and early acquisition of neural-like characteristics, potentially indicating initial stages of neurite extension. The non-confluent but stable adhesion is a sign of good cytocompatibility, demonstrating that the NGC can support the attachment and survival of differentiated hDPSCs, as well as undifferentiated hDPSCs, as previously studied [2]. This is particularly relevant in the context of peripheral nerve regeneration, where cell–scaffold interactions help direct axonal regrowth [8,69]. EDS analysis of the cell-covered regions confirmed the presence of biological material through detection of nitrogen, carbon, and oxygen, elements commonly associated with cellular membranes and protein structures [70].

In parallel, combined SEM and EDS analyses also confirmed the successful isolation of hDPSC-derived exosomes and their adhesion in the NGC. The vesicles displayed typical exosomal morphology, spherical to slightly concave profiles, with diameters ranging between 100 and 130 nm [71]. Their preserved structure and even distribution suggest good stability and compatibility with the NGC surface. Elemental mapping from zones Z1 and Z2, where exosomes were identified, revealed an organic signature comprising carbon, nitrogen, oxygen, and phosphorus, consistent with lipid bilayer components and internal nucleoproteins. In contrast, the control region Z3, without vesicles, showed an inorganic profile, dominated by silica and calcium, in line with the composition of the scaffold material or processing residues. This distinction confirms that the biological signal in Z1 and Z2 arises specifically from the exosomes, not from the substrate.

Altogether, these results validate both the morphology and biochemical composition of the exosomes and neurodifferentiated hDPSCs and confirm their interaction with the biomaterial. The ability of vesicles and cells to adhere to the scaffold while retaining structural integrity supports their application as paracrine effectors in cell-free regenerative strategies, especially in systems designed for guided nerve repair [72].

The capacity of hDPSCs to differentiate toward neurogenic lineages is of increasing interest, particularly due to their neural crest origin and associated plasticity [57]. In this study, neurogenic induction activated a transcriptional profile consistent with early neuronal and astroglial behavior. This profile is more consistent with an early or partial lineage commitment, as the capacity of MSCs to achieve full neuroglial differentiation remains debated in the literature [73]. Differentiated hDPSCs expressed upregulations in markers such as *Ascl1*, *Cd40*, *Nestin*, *Cdk5r1*, *Cript*, *Sox2*, *Cd40*, and *Cdh2*, associated with neural progenitor activation and early lineage progression [74,75,76,77,78]. The presence of *Map2*, *Dcx*, and *Syp*, typically linked to cytoskeletal remodeling, migration, and synaptic vesicle formation, respectively, suggests that the cells acquired immature neuronal characteristics, without reaching full maturation [79]. Of relevance was the expression of *Gfap* and *Aldh1l1*, two well-established astrocytic markers, detected exclusively in differentiated hDPSCs. This indicates that the induction protocol activates not only neuronal but also astroglial differentiation pathways, reflecting the natural lineage diversity observed during early neural development [79]. This dual activation may be advantageous in regenerative applications, as both neurons and glial cells contribute to successful nerve repair through trophic support, modulation of inflammation, and structural guidance [80,81].

In contrast, undifferentiated hDPSCs showed expression of *Tubb3*, *Ocln*, *Sox10*, and *Cspg4*. The presence of *Sox10* and *Cspg4*, associated with neural crest-derived progenitors and immature glial precursors, confirms the multipotent state of the undifferentiated cells [82,83,84]. *Ocln*, a tight junction protein, further supports an epithelial-like form [85]. Interestingly, *Tubb3*, typically regarded as a pan-neuronal marker, was present in the undifferentiated group but absent in differentiated cells. This may reflect dynamic regulation of tubulin isoforms during early neuronal commitment or the predominance of alternative class III β-tubulin isoforms not targeted by the primers used [79,86].

The absence of *Mpz*, *Ncam1*, *Rbfox3*, and *Olig3* in both groups suggests that the differentiation protocol induced a partial neural commitment, but not terminal maturation into specific subtypes such as myelinating glia or fully functional neurons [87,88]. This incomplete profile is not unexpected given the relatively short induction period. Future protocols may benefit from longer culture durations and more targeted lineage cues to guide differentiation toward functional subtypes relevant for peripheral nerve regeneration. The downregulation of *Gap43*, a gene associated with axonal growth and neural remodeling, further supports the incomplete activation of regenerative pathways [89]. Although often upregulated during active neurite extension, its decreased expression may reflect insufficient cytoskeletal remodeling or lack of stimuli promoting axonal outgrowth [57,90,91]. Combined with the absence of maturation markers such as *Mpz*, *Ncam1*, *Rbfox3*, and *Olig3*, these results suggest that the applied differentiation protocol induced early neuroglial commitment but did not drive full maturation toward functionally specialized neuronal or glial subtypes [57,92]. The predominance of progenitor and early-lineage markers in the transcriptomic profile aligns with a transitional phenotype, which may require prolonged induction to achieve more advanced differentiation stages [74,93,94].

The analysis of 22 neuroglial-related genes in exosomes derived from undifferentiated hDPSCs revealed a selective and passage-dependent expression pattern. Although a greater number of transcripts were detected in P7-derived exosomes, many were present at low levels, suggesting that a broader transcript distribution does not necessarily equate to higher functional potential. More relevantly, key neurogenic and glial markers such as *Tubb3*, *Gfap*, and *Mpz*, showed reduced expression in P7 compared to P4. The marked downregulation of *Tubb3* is particularly indicative of diminished neurogenic signaling, while the moderate decrease in *Gfap* and *Mpz* reflects a decline in glial and myelination-related content [95,96,97]. These findings suggest that exosomes from early-passage hDPSCs carry a transcript profile more compatible with neural repair.

Conversely, *Cd40* and *Cdk5r1* were significantly upregulated in exosomes from P7. *CD40* is involved in immune activation and may reflect a shift toward inflammatory or stress-related pathways in aging cells [98]. *Cdk5r1*, a key regulator of neuronal cytoskeletal dynamics, is also implicated in cellular stress response and synaptic function [99,100]. Some transcripts, including *Ascl1*, *Sox10*, and *Gap43*, remained relatively stable between passages, indicating partial conservation of neural-related signals despite replicative aging [101,102,103]. Interestingly, genes such as *Dcx*, *Ncam*, *Nes*, *Olig3*, *Syp*, and *Sox2* were exclusively detected in P7-derived exosomes. The consistent absence of *Aldh1l1*, *Cdh2*, *Cript*, *Cspg4*, *Ocln*, *Rbfox3*, and *Neurod1* in exosomes from both early- and late-passage cells suggests that these transcripts are either not expressed at detectable levels or are not selectively incorporated into vesicles under basal conditions in undifferentiated hDPSCs. This supports the broader understanding that exosomal mRNA cargo does not merely mirror the cellular transcriptome, but results from active and regulated packaging mechanisms, likely influenced by cell state, passage number, and intracellular stress [104].

In summary, these findings confirm that replicative age modulates the exosomal transcriptome, with early-passage hDPSCs generating vesicles enriched in canonical neuroglial transcripts, and late-passage cells exhibiting a shift toward immune or stress-related signals. This reinforces the importance of controlling passage number when producing exosome-based therapies aimed at neuroregeneration. Overall, the results show that neurogenic induction triggers early neuroglial gene expression in hDPSCs, and that exosomes from early-passage cells carry mRNA profiles that may support nerve regeneration [79,105]. The observed impact of passage number on both cellular and exosomal RNA content emphasizes the importance of standardized expansion protocols and suggests that exosome profiling could serve as a useful quality control tool in the development of regenerative therapies [34].

The neurite outgrowth assay provided a reproducible and sensitive platform for evaluating neuritic extension under defined experimental conditions. The results demonstrated that hDPSCs respond dynamically to both permissive and inhibitory factors applied to the basal membrane surface. Membranes coated with laminin elicited robust neurite extension, in agreement with previous literature describing laminin as a pro-neurogenic extracellular matrix protein [106,107]. The pattern of neurite staining observed on the basal surface supports its efficacy in promoting axonal-like outgrowth, likely via integrin-mediated cytoskeletal signaling pathways [108,109].

The near absence of neuritic structures in the BSA-coated condition confirmed the biological inertness of BSA in this context and validated its role as a negative control [110]. Moreover, the lack of diffuse or non-specific signal in these membranes supports the assay’s capacity to selectively visualize membrane-translocating neuritic processes.

To assess the inhibitory modulation of neurite outgrowth, the microtubule-depolymerizing agent nocodazole was used. Nocodazole is a synthetic benzimidazole that binds to β-tubulin and disrupts microtubule polymerization, thereby impairing cytoskeletal dynamics essential for neurite formation and elongation [111,112]. This compound has been widely used in neurobiological studies as a tool to investigate the dependence of neurite extension on microtubule integrity [113,114]. In the present study, nocodazole was applied at two different time points to induce partial and complete inhibition of neurogenesis. The observed time-dependent suppression of neurite formation is consistent with the critical role of stable microtubules in supporting neuritic protrusion and guidance [115,116,117]. To confirm the role of microtubule stability in neurite formation, nocodazole was applied at 250 ng/mL either from the beginning of differentiation or after a 24 h priming period. When nocodazole was present from time zero, neurite extension was severely impaired, reflecting the critical role of early microtubule polymerization in neurogenesis [118]. Delayed exposure to nocodazole (after 24 h of differentiation) resulted in a milder reduction in neurite staining. This suggests that once neurites have initiated and partially matured, they may retain some structural stability or partial resistance to microtubule depolymerization, at least at the tested concentration. This finding aligns with reports that microtubule bundles in stabilized neurites exhibit increased resilience compared to dynamic, newly formed projections [119,120,121].

Quantitatively, absorbance measured at 570 nm showed a significant increase in the laminin-coated group, supporting its role as a neuroinductive substrate. In contrast, BSA-coated membranes exhibited minimal absorbance, confirming the absence of neurite formation. Nocodazole treatment from the beginning of differentiation markedly reduced absorbance values, indicating early inhibition of microtubule dynamics. When nocodazole was added after 24 h, absorbance levels remained high, suggesting that pre-established neurites retain partial resistance to microtubule depolymerization. These data support the reliability of the assay in distinguishing permissive from inhibitory conditions for neurite extension.

The quantification of total protein revealed distinct patterns depending on both the differentiation status and passage number of the hDPSCs. In neurodifferentiated cells, there was a decrease in protein concentration from P4 to P7, suggesting a decline in global secretory activity associated with replicative aging. However, an opposite trend was observed in undifferentiated cells: both the CM and exosome fractions displayed higher total protein levels at P7 compared to P4, with the CM from undifferentiated P7 cells exhibiting the highest value among all the groups. This increase may reflect changes in cell metabolism, stress-related responses, or compensatory secretion mechanisms in late passages [122], although further studies are required to clarify the underlying causes. Notably, in all conditions, the CM consistently showed higher protein content than the corresponding exosome fraction, confirming that the soluble secretome encompasses a broader range of bioactive molecules beyond the vesicles [123]. These findings highlight the importance of considering both differentiation state and passage number when analyzing the secretory profile of hDPSCs and designing therapeutic strategies. The divergent behaviors observed emphasize the need for careful selection and standardization of culture parameters in the development of secretome- or exosome-based regenerative products.

These results collectively reinforce the notion that the therapeutic potential of hDPSC-derived products is not fixed but evolves with culture conditions and cellular aging. The neurogenic environment amplifies the regenerative signature of both the CM and exosomal fractions, particularly at early passages. Importantly, the sensitivity of hDPSCs to microenvironmental modulation, demonstrated by their response to both neuroinductive cues and cytoskeletal disruption, underscores their plasticity. These findings highlight the need to rigorously define and control in vitro parameters such as passage number, induction timing, and concentration protocols to harness the full regenerative potential of hDPSCs. Products derived from this cell lineage, when prepared under optimized conditions, represent a promising strategy for peripheral nerve repair, particularly in combination with NGC, offering a reproducible, cell-free approach with strong translational relevance. Nonetheless, the variability observed across the different analyses suggests that additional strategies may be required to further enhance the consistency and efficacy of hDPSC-derived products. One promising technique could involve the application of priming protocols to precondition hDPSCs, aiming to achieve a more uniform and enhanced regenerative profile [124,125]. Such modulation could help overcome variations seen in secretory, transcriptomic, and functional assays, ultimately strengthening the reliability and translational potential of these products for neuroregenerative applications.

Future studies should focus on validating these regenerative effects in vivo. Animal models of PNI are an essential step to determine whether the CM and exosomal cargo of early-passage hDPSCs can support functional recovery, including axonal regrowth and remyelination [8,10,126]. It will also be important to test the combination of hDPSC with clinically approved NGCs, as this could better reflect their therapeutic applicability [2]. Clinically, hDPSCs are an attractive source because dental pulp can be obtained with minimal invasiveness, and the cells display a favorable immunological profile [127]. Incorporating the hDPSC-CM or exosomes into biocompatible scaffolds may further facilitate the development of reproducible therapies for peripheral nerve repair [8,11,128]. In this context, our findings highlight the translational potential of hDPSC-CM and hDPSC-derived exosomes, particularly those obtained from early-passage cells, which were enriched in regenerative factors relevant to peripheral nerve repair. This supports their potential as candidates for cell-free and standardized therapeutic approaches. While the present study is preclinical and limited to in vitro assays, it provides a solid foundation for subsequent in vivo validation, safety assessment, and regulatory studies aimed at exploring clinically applicable viable products.

## 4. Materials and Methods

### 4.1. Preparation of hDPSCs and Conditioned Medium

The isolation, expansion and characterization methodologies for hDPSCs were previously described in detail by our research group [2,9,129,130,131]. A commercially available hDPSC primary cell line (AllCells, LLC, Alameda, CA, USA; Cat. DP0037F, Lot DPSC090411–01) was used, which has been previously validated by the supplier regarding its primary cell properties. Conditioned medium derived from hDPSCs was produced using previously established protocols, and extensively characterized for its periphery injury healing potential, as described in Bruna et al. [1,2].

Briefly, hDPSCs were cultured under standard conditions, washed, and incubated in unsupplemented DMEM/F12 GlutaMAX^™^ (Thermo Fisher Scientific, Waltham, MA, USA) for 48 h. After this period, the CM was collected and centrifuged to remove cellular debris, followed by filtration. At this stage, two types of samples were prepared: (i) unconcentrated CM (1×), which was aliquoted and stored at −20 °C, and (ii) concentrated CM (5×), which was further processed by centrifugal filtration devices to obtain a fivefold concentration before storage at −20 °C. Both 1× and 5× CM were subsequently analyzed using the Luminex^™^ 200 system (Luminex, Austin, TX, USA) by Eve Technologies Corp. (Calgary, AB, Canada) to identify specific biomarkers. Concentration values obtained from the multiplex assay are expressed in pg/mL.

In this study, the use of neurodifferentiated hDPSCs was introduced to compare their CM with that of undifferentiated cells. Additionally, exosomes were isolated from hDPSCs to investigate their content profile. These novel approaches allow a broader understanding of the differences in bioactive factors released by these cell populations.

For the experimental setup, different groups of hDPSCs and their derived products were analyzed. The groups included:

- Undifferentiated hDPSCs at P7 and P4, both with unconcentrated CM (1×).
- Neurodifferentiated hDPSCs at P4 and P7 with unconcentrated CM (1×).
- Neurodifferentiated hDPSCs at P4 and P7 with concentrated CM (5×).
- Exosomes isolated from hDPSCs at P4 and P7 with unconcentrated CM (1×).
- Exosomes isolated from hDPSCs at P4 and P7 with concentrated CM (5×).

### 4.2. Analysis of hDPSC Conditioned Medium

The CM was analyzed to identify key chemokines and growth factors secreted by hDPSCs. This study included both early-passage (P4) and late-passage (P7) hDPSCs to compare their secretory profiles. Additionally, CM from neurodifferentiated hDPSCs and exosomes isolated from hDPSCs were explored, extending our previous investigations, including the evaluation of two different concentrations [2].

Luminex xMAP technology was employed for the multiplexed quantification of 48 human cytokines, chemokines, and growth factors. The analysis was performed using Eve Technologies’ Human Cytokine Panel A 48-Plex Discovery Assay^®^ (MilliporeSigma, Burlington, MA, USA) following the manufacturer’s protocol. The 48-plex panel included a broad range of biomarkers relevant to peripheral nerve regeneration, with sensitivities ranging from 0.14 to 50.78 pg/mL.

A comprehensive explanation of each biomarker and its role in this study is detailed in Bruna et al. [2].

### 4.3. Exosome Isolation from Cell Culture Media

Exosomes were isolated from hDPSCs culture media using the Total Exosome Isolation from cell culture media Kit (Thermo Fisher Scientific, Cat. No. 4478359), following the manufacturer’s instructions and another study from the group in the subject [132]. Briefly, the conditioned culture media were first centrifuged at 2000× *g* for 30 min to remove cells and debris. The supernatant was carefully transferred to a new tube, avoiding pellet disturbance. Exosomes were then precipitated by adding 0.5 volumes of the Total Exosome Isolation reagent to the cleared media, mixing thoroughly by vortexing, and incubating overnight at 2 °C to 8 °C. The mixture was then centrifuged at 10,000× *g* for 1 h at 4 °C. The supernatant was discarded, and the exosome pellet was resuspended in 1× PBS to the desired volume. Isolated exosomes were stored at 2 °C to 8 °C for short-term use or at ≤−20 °C for long-term storage.

### 4.4. Neurogenic Differentiation Assay

For neurogenic differentiation, 4 × 10^3^ cells/cm^2^ hDPSCs were seeded into a 12-well plate. The plate was maintained under standard conditions until cells reached 70–80% confluency. Media was removed from all the wells, and the neurogenic differentiation medium (MSC Differentiation Medium, PromoCell^®^, Heidelberg, Germany) was allocated in 8 wells, while the remaining 2 wells were used as controls and maintained with the usual culture medium. Cells were maintained under differentiation for 4 days, and media were changed every 48 h. A comprehensive explanation of neurogenic differentiation is detailed in Bruna et al. [2].

### 4.5. Scanning Electron Microscopy

Following the analysis of hDPSCs-CM and derived exosomes, a scanning electron microscopy analysis and energy-dispersive X-ray spectroscopy were conducted. This analysis used a high-resolution (Schottky) Environmental Scanning Electron Microscope equipped with X-ray Microanalysis and Electron backscattered diffraction (FEI Quanta 400 FEG ESEM (FEI Company, Hillsboro, OR, USA) EDAX Genesis X4M (EDAX Inc., Mahwah, NJ, USA)). The microscope operated in high vacuum mode at an acceleration mode of 15 kV SEM. Reaxon^®^ NGCs were cut transversely to fit the diameter of a well in a 24-well plate and then longitudinally. Each half-NGC was placed inside a well, and P4 hDPSCs were seeded at a density of 6000 cells/cm^2^ with basal culture medium on the internal and external surface of the cut NGCs. The cells were cultured for 216 h with medium changes every 2–3 days. The undifferentiated SEM was previously performed [2].

In this study, after this initial culture period, neurodifferentiation was induced within the NGC by replacing the culture medium with a neurogenic differentiation medium. This approach allowed assessment of whether hDPSCs could differentiate while maintaining adhesion to the NGC structure. Additionally, exosomes were directly seeded onto the Reaxon^®^ scaffold and left at room temperature for one hour before undergoing the same fixation protocol as the neurodifferentiated cells. This distinct approach was necessary due to their higher sensitivity, allowing us to assess their adhesion and morphological characteristics within the scaffold.

Following this process, both neurodifferentiated cells and exosomes underwent the same washing and fixation procedures. Wells were washed three times with 0.1 M HEPES buffer (Merck^®^, Darmstadt, Germany, PHG0001). Cells on the NGCs’ inner surface were fixed with 2% buffered glutaraldehyde (Merck^®^, G7651) and left overnight in the refrigerator to ensure a slower process, while the exosomes only stayed one hour in this condition. Afterward, cells and exosomes were washed in three cycles of five minutes with 0.1 M HEPES buffer with gentle agitation. Samples were then dehydrated through a graded ethanol series (50%, 70%, 90%, 99%), with each concentration applied 2–3 times for 10–15 min. Finally, samples were infiltrated with a graded series of hexamethyldisilazane (HMDS) (Merck^®^, 440191) in ethanol for 15 min and incubated with HMDS alone for another 15 min. After removing HMDS, plates were left overnight in a laminar flow chamber for complete evaporation. Prior to SEM and EDS analysis, samples were coated with gold/palladium for 80 s using a 15 mA current.

### 4.6. RT-PCR

To assess the expression of specific genes in hDPSCs, both in their undifferentiated state and after neurogenic differentiation, RT-PCR was performed. In addition, exosomes derived from hDPSCs were also assessed.

#### 4.6.1. RNA Isolation and cDNA Synthesis from hDPSCs

RNA was extracted from hDPSCs at P4 in both undifferentiated conditions and following neurogenic differentiation, which was induced according to previously established protocols [2,133]. The isolation process was carried out using the Aurum^™^ Total RNA Mini Kit (Bio-Rad Laboratories^®^, Hercules, CA, USA), following the manufacturer’s guidelines. Briefly, a pellet containing 2 × 10^6^ cells, both differentiated (after 72 h of induction) and undifferentiated, was lysed using a lysis buffer. DNA contamination was eliminated using DNase I treatment, and the RNA was subsequently eluted in 80 μL of elution buffer. The purified RNA was stored at −80 °C until further use.

Before proceeding with cDNA synthesis, RNA purity and concentration were evaluated using UV spectrophotometry. Absorbance ratios at A260/A280 (to assess protein contamination) and A260/A230 (to detect potential contamination with phenol, polysaccharides, or chaotropic salts) were measured using a NanoDrop^™^ One Microvolume UV-Vis Spectrophotometer (Thermo Scientific^™^). Acceptable purity values were considered within the range of 2.0–2.2 for A260/A280 and 1.8–2.2 for A260/A230 [8].

First-strand cDNA synthesis was performed using 4 μL of total RNA in a final reaction volume of 20 μL, following the instructions provided in the iScript^™^ cDNA Synthesis Kit (Bio-Rad Laboratories^®^). The reaction mixture was incubated in a thermal cycler (T100^™^ Thermal Cycler, Bio-Rad Laboratories^®^) under the time and temperature conditions specified by the manufacturer.

#### 4.6.2. RNA Isolation and cDNA Synthesis from Exosomes Derived from hDPSCs

Total RNA was isolated from hDPSC-derived exosomes using the Total Exosome RNA and Protein Isolation Kit (Thermo Fisher Scientific, Cat# 4478545), following the manufacturer’s protocol. Briefly, samples were adjusted to a final volume of 200 μL with 1XPBS if necessary, followed by lysis and phase separation using Acid-Phenol/Chloroform. The aqueous phase was collected, and RNA was precipitated with ethanol before being passed through a filter cartridge via centrifugation. The retained RNA was washed with miRNA Wash Solution 1 and Wash Solution 2/3, followed by a final centrifugation step to remove residual contaminants. RNA was eluted in 50 μL of preheated (95 °C) Elution Solution or nuclease-free water and stored at ≤−20 °C until further use.

Before proceeding with cDNA synthesis, RNA concentration and purity were assessed using UV spectrophotometry on a Nanodrop device (Implen GmbH, Isaza^®^, Munich, Germany). Purity was evaluated through the A260/A280 and A260/A230 absorbance ratios, which serve as indicators of protein contamination and the presence of polysaccharides, phenol, or chaotropic agents, respectively. Samples were considered acceptable if the A260/A280 ratio ranged between 2.0 and 2.2, and the A260/A230 ratio fell within 1.8 to 2.2.

First-strand cDNA synthesis was performed using the iScript^™^ cDNA Synthesis Kit (Bio-Rad Laboratories^®^) according to the manufacturer’s instructions, with 4 μL of total RNA in a final reaction volume of 20 μL. The reaction was carried out in a T100^™^ Thermal Cycler (Bio-Rad Laboratories^®^) under standard thermal conditions recommended by the manufacturer.

### 4.7. Quantitative RT-PCR Assay

The quantitative RT-PCR assay was performed using the CFX96 Touch^™^ Real-Time PCR Detection System (Bio-Rad Laboratories^®^) under standard reaction conditions. The amplification process was carried out with iTaq^™^ Universal SYBR Green Supermix (Bio-Rad Laboratories^®^), following the manufacturer’s protocol. A total of 22 genes related to neuroglial markers were analyzed, encompassing markers of both immature and mature neurons, as well as glial cells at various maturation stages. To ensure the reliability of the qRT-PCR assays, RNA purity and integrity were verified by NanoDrop^™^ spectrophotometry prior to cDNA synthesis. Amplification specificity was assessed by melting curve analysis, which consistently revealed a single peak without secondary products.

Markers associated with glial cells included Aldh1l1, CD40, Cdh2, Cspg4, GAP43, GFAP, MPZ, NCAM, Nes, Ocln, Olig3, Sox10, and Sox2. Neuronal markers included Ascl1, Dcx, MAP2, NeuroD1, Rbfox3, Syn, Cdk5r1, Cript, Tubb3, and GAP43 (also classified as a glial marker). β-Actin was used as the reference housekeeping gene.

A 96-well PrimePCR Custom Plate (Bio-Rad Laboratories^®^) was prepared, containing predesigned primers for the selected 22 genes to enable their expression analysis. The plate was processed in a real-time PCR system, where two sets of primers were used to assess gene expression levels in both undifferentiated and differentiated P4 hDPSCs, as well as in exosomes derived from hDPSCs. The reaction mix targeting the 22 genes underwent temperature cycling according to the manufacturer’s specifications. Upon completion, gene expression levels were analyzed, including an evaluation of melting curves to verify product specificity.

Threshold cycle (Ct) values were interpreted as follows:

- Ct < 29: Strong positive reaction, indicating a high abundance of the target nucleic acid.
- 30 < Ct < 39: Moderate gene expression, with a detectable but lower quantity of the target nucleic acid.
- Ct > 39: Weak signal, suggesting minimal target nucleic acid presence or possible environmental contamination.

To compare gene expression between the two groups, ΔCt values were calculated using the formula:

$$\Delta Ct=Ct\left(target\;gene\right)-Ct\left(housekeeping\;gene\right)$$

Fold changes between differentiated and undifferentiated cells were determined using the ΔΔCt method, where

$$\Delta \Delta Ct=\Delta Ct\left(differentiated\right)-\Delta Ct\left(undifferentiated\right)$$

Relative quantification (RQ) of gene expression was calculated as follows:

(1) $$RQ=2^{-\left(\Delta \Delta Ct\right)}$$

Genes were classified as downregulated when RQ values were <0.5 and upregulated when RQ values were >2.

### 4.8. Neurite Outgrowth Assay

Neurite outgrowth was assessed using the Neurite Outgrowth Assay Kit (NS220, Chemicon^®^, Merck KGaA, Darmstadt, Germany), following the manufacturer’s protocol. hDPSCs were cultured in appropriate growth medium until reaching 70–80% confluence. Cells were then primed by replacing the culture medium with DMEM/F12 GlutaMAX Supplement (Gibco^®^, Waltham, MA, USA) containing 1% FBS and 1% penicillin/streptomycin, followed by a 24 h incubation to arrest proliferation.

For membrane preparation, Millicell inserts (1 µm pore size, included in the kit) were coated with Type I Collagen (10 µg/mL, Corning^®^, Corning, NY, USA) diluted in 1X PBS with Ca^2+^/Mg^2+^ (Gibco^®^). Inserts were incubated at 37 °C for 2 h before cell seeding. Control wells were coated with Bovine Serum Albumin (BSA, Gibco^®^) instead of collagen.

hDPSCs were detached using 0.05% trypsin-EDTA and resuspended in PromoCell^®^ differentiation medium at a density of 1–2 × 10^6^ cells/mL. Inserts were transferred to wells containing 600 µL of differentiation medium, and 100 µL of the cell suspension was added on top of each insert. After 15 min at room temperature for uniform distribution, cultures were incubated at 37 °C with 5% CO_2_ for 48 h to allow neurite extension.

To assess the effects of nocodazole, differentiation conditions included: control (BSA-coated wells), with differentiation without nocodazole; standard differentiation, with cells differentiated in PromoCell^®^ medium without nocodazole; nocodazole 0 h, with cells treated with 250 ng/mL nocodazole (Sigma-Aldrich^®^, St. Louis, MO, USA) from the beginning of differentiation; and nocodazole 24 h, with cells differentiated for 24 h followed by the addition of 250 ng/mL nocodazole for another 24 h.

Neurite extensions were fixed in methanol (−20 °C, 20 min) and stained using the Neurite Stain Solution (NS220, Chemicon^®^) for 15–30 min. After washing with PBS, cell bodies were removed using flattened cotton swabs. Stained neurites were extracted using Neurite Stain Extraction Buffer (NS220, Chemicon^®^), and absorbance was measured at 562 nm in a spectrophotometer for quantification.

### 4.9. Total Protein Quantification

Exosomal protein concentration was determined using the Pierce^™^ Dilution-Free™ Rapid Gold BCA Protein Assay (Thermo Fisher Scientific, Cat. No. A55860), following the manufacturer’s protocol. The working reagent was prepared by mixing Dilution-Free^™^ Rapid Gold BCA Reagent A with Reagent B in a 50:1 ratio. Then, 10 µL of each sample was added to a 96-well microplate in triplicate, followed by 200 µL of the WR. The plate was mixed thoroughly on a plate shaker for 30 s and incubated at room temperature for 5 min. In an alkaline environment, bicinchoninic acid (BCA) reacts with cuprous ions (Cu^+^), which are generated through the reduction of cupric ions (Cu^2+^) by peptide bonds in proteins [134,135]. This reaction leads to the formation of a stable, water-soluble complex with a characteristic purple coloration, which can be quantified spectrophotometrically. Absorbance was measured at 450 nm using a microplate reader, with a secondary measurement at 570 nm for background correction.

A standard curve was generated using the BSA standards, and the protein concentrations of the exosomal samples and CM of differentiated and undifferentiated hDPSCs were calculated based on the average absorbance of the triplicates.

### 4.10. Statistical Analysis

Statistical analysis was carried out using GraphPad Prism version 6.00 for Mac OS x (GraphPad Software, La Jolla, CA, USA). Data were expressed as mean ± SEM when appropriate. Group comparisons were conducted using parametric tests. The normality of the data was assessed using the Shapiro–Wilk test. For comparisons between two groups, unpaired *t*-tests were used, while differences involving multiple groups or factors were evaluated using two-way ANOVA, followed, when appropriate, by Tukey’s multiple comparisons post hoc test. A value of *p* < 0.05 is considered statistically significant. Significance of the results is shown according to *p* values by the symbol (*), (*) corresponding to 0.01 ≤ *p* < 0.05, (**) to 0.001 ≤ *p* < 0.01, (***) to 0.0001 ≤ *p* < 0.001 and (****) to *p* < 0.0001.

## 5. Conclusions

This study demonstrates that neurogenic differentiation, passage number, and CM concentration critically influence the secretory and vesicular profiles of hDPSCs. Early-passage, neurodifferentiated hDPSCs produce CM and exosomes enriched in regenerative and immunomodulatory factors, supporting key processes such as angiogenesis, neuroprotection, and immune regulation. Although replicative aging alters this profile, certain functional components are selectively maintained, indicating a dynamic but partially conserved paracrine capacity.

Importantly, the isolation and characterization of hDPSC-derived exosomes confirmed their structural integrity, typical size distribution, and biochemical identity, as well as their compatibility with a nerve guidance conduit. These findings validate not only the feasibility of harvesting exosomes from this cell source but also their potential role as stable, cell-free effectors of regeneration.

Collectively, the findings support the application of hDPSC-derived products as a promising strategy for peripheral nerve repair. Their ability to interact effectively with NGCs such as Reaxon^®^ further reinforces their translational value and highlights optimal production conditions for future clinical applications.

The identification of these optimal conditions is particularly relevant for in vivo studies and for the development of commercially viable therapeutic products, since each of these factors directly influences the quality of nerve regeneration and the final clinical outcomes. Future studies should also address the secretion of specific neurotrophins not covered in the present multiplex approach, to complement the broad profiling performed here and provide deeper insights into the neuroregenerative potential of hDPSCs.

## Figures and Tables

**Table 1:** The neurodifferentiated hDPSCs-CM analysis with mean concentration values for each biomolecule in P4 and P7 in both concentrations (×1) and (×5) (mean ± standard error of the mean (SEM)), expressed in pg/mL.

Biomolecule	Mean ± SEM (P4) (×1)	Mean ± SEM (P4) (×5)	Mean ± SEM (P7) (×1)	Mean ± SEM (P7) (×5)
EGF	1.40 ± 0.00	1.77 ± 0.77	2.84 ± 0.63	0.70 ± 0.00
Eotaxin	41.66 ± 0.65	148.06 ± 3.26	4.93 ± 0.49	2.90 ± 1.00
FGF-2	31.24 ± 4.03	73.33 ± 1.42	40.83 ± 3.00	108.31 ± 5.20
FLT-3L	0.61 ± 0.11	1.42 ± 0.13	0.23 ± 0.06	0.25 ± 0.00
Fractalkine	5.44 ± 4.54	8.27 ± 3.21	7.06 ± 7.98	2.83 ± 0.00
Groα	1.26 ± 0.21	2.03 ± 0.06	14.93 ± 0.41	25.46 ± 0.91
IFN α 2	2.96 ± 0.45	1.98 ± 0.64	nd	nd
IFNγ	0.3 ± 0.03	1.01 ± 0.09	0.35 ± 0.10	1.06 ± 0.03
IL-1α	0.29 ± 0.06	0.46 ± 0.12	nd	nd
IL-1b	0.71 ± 0.49	0.81 ± 0.55	0.76 ± 0.00	0.72 ± 0.36
IL-1RA	0.21 ± 0.03	0.25 ± 0.06	nd	0.19 ± 0.13
IL-4	0.51 ± 0.01	1.48 ± 0.09	0.58 ± 0.11	1.58 ± 0.06
IL-6	95.44 ± 2.04	254.07 ± 5.16	6.53 ± 0.19	21.07 ± 0.44
IL-8	11.7 ± 0.51	37.67 ± 3.48	35.89 ± 1.83	102.60 ± 7.78
IL-9	2.36 ± 0.48	2.13 ± 0.00	nd	0.64 ± 0.00
IL-10	0.16 ± 0.03	nd	nd	nd
IL-12p40	1.25 ± 1.21	2.39 ± 3.06	2.11 ± 0.00	nd
IL-13	6.82 ± 1.10	15.56 ± 1.74	9.13 ± 0.92	15.93 ± 1.77
IL-15	4.07 ± 0.17	14.02 ± 0.87	1.37 ± 0.17	15.93 ± 1.77
IL-18	0.08 ± 0.02	nd	nd	nd
IL-22	21.07 ± 2.09	43.86 ± 1.52	17.17 ± 2.32	38.18 ± 4.45
IL-27	2.01 ± 0.00	4.29 ± 4.59	5.83 ± 4.58	7.21 ± 4.73
IP-10	2.77 ± 0.31	1.91 ± 0.50	5.89 ± 0.21	4.60 ± 0.21
M-CSF	50.78 ± 3.15	156.36 ± 9.17	41.92 ± 2.91	128.81 ± 12.65
MCP-1	nd	nd	114.2 ± 5.22	428.88 ± 12.34
MCP-3	76.98 ± 1.59	217.32 ± 10.82	3.57 ± 0.69	4.11 ± 0.66
MIG	0.18 ± 0.05	nd	nd	nd
MIP	nd	nd	nd	4.02 ± 0.65
PDGF-AA	50.84 ± 2.28	192.09 ± 5.22	1.29 ± 0.21	2.92 ± 0.28
PDGF-AB/BB	25.45 ± 3.93	28.45 ± 6.69	33.69 ± 8.64	27.52 ± 5.32
TGFα	0.22 ± 0.15	nd	nd	0.23 ± 0.04
TNFα	0.75 ± 0.00	1.11 ± 0.60	nd	14.90 ± 1.49
TNFβ	5.49 ± 0.56	15.2 ± 1.30	5.79 ± 0.39	1.80 ± 0.07
RANTES	3.27 ± 0.35	7.00 ± 0.27	0.95 ± 0.37	356.41 ± 9.34
VEGF-A	98.89 ± 3.39	331.81 ± 4.57	99.72 ± 5.17	0.55 ± 0.00
sCD40	nd	3.27 ± 2.79	2.72 ± 3.41	nd

nd—nondefined.

**Table 2:** hDPSC-derived exosome analysis with mean concentration values for each biomolecule in P4 and P7 in both concentrations (×1) and (×5) (mean ± (SEM) expressed in pg/mL.

Biomolecule	Mean ± SEM (Exosomes P4) (×1)	Mean ± SEM (Exosomes P4) (×5)	Mean ± SEM (Exosomes P7) (×1)	Mean ± SEM (Exosomes P7) (×5)
EGF	0.70 ± 0.00	nd	nd	0.91 ± 0.00
Eotaxin	0.60 ± 0.65	0.60 ± 0.00	0.41 ± 0.00	0.86 ± 0.00
FGF-2	0.08 ± 0.00	nd	nd	nd
FLT-3L	nd	0.16 ± 0.09	0.07 ± 0.04	0.07 ± 0.04
Fractalkine	nd	3.3 ± 0.00	nd	nd
IL-1α	nd	0.12 ± 0.07	nd	nd
IL-1b	nd	0.34 ± 0.23	nd	0.06 ± 0.00
IL-1RA	0.03 ± 0.00	0.12 ± 0.09	nd	0.19 ± 0.13
IL-5	0.05 ± 0.01	0.04 ± 0.01	0.04 ± 0.01	0.04 ± 0.00
IL-8	0.01 ± 0.00	0.06 ± 0.05	0.01 ± 0.00	0.01 ± 0.00
IL-9	nd	0.64 ± 0.00	nd	nd
IL-10	0.03 ± 0.00	0.16 ± 0.03	0.09 ± 0.03	0.08 ± 0.05
IL-12p40	nd	0.09 ± 0.00	nd	nd
IL-13	2.76 ± 1.17	2.89 ± 1.86	2.44 ± 1.42	2.29 ± 1.12
IL-15	0.09 ± 0.07	0.19 ± 0.06	0.32 ± 0.11	0.04 ± 0.00
IL-18	nd	nd	nd	nd
IL-22	0.41 ± 0.00	nd	nd	nd
IL-27	3.60 ± 0.88	nd	nd	nd
M-CSF	1.78 ± 0.54	1.78 ± 0.54	12.58 ± 0.00	5.66 ± 0.00
MCP-3	1.76 ± 0.90	1.77 ± 0.32	1.34 ± 0.18	0.27 ± 0.00
MIG	0.14 ± 0.09	0.08 ± 0.00	0.07 ± 0.05	0.12 ± 0.05
MIP	2.10 ± 3.07	1.31 ± 0.00	nd	nd
PDGF-AA	0.38 ± 0.27	0.40 ± 0.18	0.24 ± 0.14	0.16 ± 0.14
PDGF-AB/BB	21.07 ± 10.62	21.45 ± 2.77	12.84 ± 9.70	17.06 ± 10.61
TNFα	0.13 ± 0.01	0.13 ± 0.02	0.12 ± 0.01	0.13 ± 0.01
TNFβ	0.42 ± 0.31	0.92 ± 0.25	0.56 ± 0.38	0.63 ± 0.54
RANTES	0.17 ± 0.00	0.13 ± 0.06	nd	nd
sCD40	nd	0.55 ± 0.00	nd	nd

**Table 3:** Summary of the effects of neurodifferentiation, passage number, and concentration on key biomarkers in hDPSC-CM and their main biological roles.

Cytokine/Factor	P4	P7	Function in Nerve Regeneration
FGF-2	✓	✓	Angiogenesis, Schwann cell proliferation, axon outgrowth
VEGF-A	✓	—	Angiogenesis, neuroprotection
PDGF-AA	✓	—	Schwann cell support, tissue remodeling
IL-6	✓	—	Inflammation resolution, glial modulation
IL-8	✓	✓	Angiogenesis, chemotaxis
IL-22	✓	✓	Tissue protection, neurotrophic signaling
M-CSF	✓	✓	Macrophage activation and polarization
MCP-3	✓	—	Monocyte/macrophage recruitment
MCP-1	—	✓	Monocyte chemotaxis, immune modulation
Eotaxin	✓	—	Chemokine signaling, potential glial effects
RANTES	—	✓	T-cell chemotaxis, axon growth, remyelination

**Table 4:** Ct, ΔCt, ΔΔCt, and RQ values for all genes under study for hDPSCs, undifferentiated and after neurogenic differentiation. ↑ = upregulated; ↓ = downregulated; nd = nondefined.

	Undifferentiated		Differentiated				
Target Gene	Ct Average DPSCs	ΔCt DPSCs	Ct Average DPSCs	ΔCt DPSCs	ΔΔCt	RQ	Regulation
*Aldh1l1*	nd	nd	33.24 ± 0.02	13.14	nd	nd	nd
*Ascl1*	33.66 ± 1.33	14.36	31.78 ± 1.19	11.68	−2.68	6.41	↑
*Cd40*	24.32 ± 0.28	5.02	15.4 ± 0.00	−4.70	−9.70	843.36	↑
*Cdh2*	24.85 ± 0.52	5.55	22.63 ± 0.07	2.53	−3.02	8.11	↑
*Cdk5r1*	30.47 ± 0.05	11.17	29.52 ± 0.73	9.42	−1.75	3.36	↑
*Cript*	25.59 ± 0.01	6.29	23.08 ± 0.28	2.98	−3.31	9.92	↑
*Cspg4*	28.4 ± 0.00	9.10	nd	nd	nd	nd	nd
*Dcx*	nd	nd	37.35 ± 0.64	17.25	nd	nd	nd
*Gap43*	36.05 ± 0.91	16.75	38.75 ± 0.28	18.65	1.90	0.27	↓
*Gfap*	nd	nd	36.3 ± 0.13	16.20	nd	nd	nd
*Map2*	nd	nd	30.08 ± 0.67	9.98	nd	nd	nd
*Mpz*	nd	nd	nd	nd	nd	nd	nd
*Ncam*	nd	nd	nd	nd	nd	nd	nd
*Nes*	25.91 ± 0.75	6.61	20.24 ± 0.70	0.14	−6.47	88.65	↑
*Neurod1*	34.84 ± 0.52	15.54	36.56 ± 0.34	16.46	0.92	0.53	N
*Ocln*	24.51 ± 0.00	5.21	nd	nd	nd	nd	nd
*Olig3*	nd	nd	nd	nd	nd	nd	nd
*Rbfox3*	nd	nd	nd	nd	nd	nd	nd
*Sox10*	35.3 ± 0.13	16.00	nd	nd	nd	nd	nd
*Sox2*	33.21 ± 0.00	13.91	29.25 ± 0.20	9.15	−4.76	37.10	↑
*Syp*	nd	nd	26.68 ± 0.11	6.58	nd	nd	nd
*Tubb3*	38.78 ± 0.00	19.48	nd	nd	nd	nd	nd

**Table 5:** Ct, ΔCt, ΔΔCt, and RQ values for all genes under study for hDPSC exosomes. ↑ = upregulated; N = normal. ↓ = downregulated; nd = nondefined.

	hDPSCs Exosomes P4		hDPSCs Exosomes P7				
Target Gene	Ct Average Exo P4	ΔCt Exo P4	Ct Average Exo P7	ΔCt Exo P7	ΔΔCt	RQ	Regulation
*Aldh1l1*	nd	nd	nd	nd	nd	nd	nd
*Ascl1*	35.31 ± 0.00	16.01	36.41 ± 0.37	16.31	0.30	0.81	N
*Cd40*	39.27 ± 0.23	19.97	36.23 ± 1.46	16.13	−3.84	14.32	↑
*Cdh2*	nd	nd	nd	nd	nd	nd	nd
*Cdk5r1*	36.33 ± 0.00	17.03	35.05 ± 0.06	14.95	−2.08	4.23	↑
*Cript*	nd	nd	nd	nd	nd	nd	nd
*Cspg4*	nd	nd	nd	nd	nd	nd	nd
*Dcx*	nd	nd	36.74 ± 0.00	16.64	nd	nd	nd
*Gap43*	39.28 ± 0.50	19.98	39.15 ± 0.36	19.05	−0.93	1.91	N
*Gfap*	39.93 ± 0.00	20.63	38.92 ± 0.00	18.82	1.81	0.29	↓
*Map2*	37.11 ± 0.00	17.81	nd	nd	nd	nd	nd
*Mpz*	39.28 ± 0.50	19.98	37.25 ± 0.81	17.15	2.83	0.14	↓
*Ncam*	nd	nd	36.18 ± 0.00	16.08	nd	nd	nd
*Nes*	nd	nd	35.85 ± 0.64	15.75	nd	nd	nd
*Neurod1*	nd	nd	nd	nd	nd	nd	nd
*Ocln*	nd	nd	nd	nd	nd	nd	nd
*Olig3*	nd	nd	38.09 ± 1.40	17.99	nd	nd	nd
*Rbfox3*	nd	nd	nd	nd	nd	nd	nd
*Sox10*	36.38 ± 0.00	17.08	37.79 ± 0.00	17.69	−0.61	1.53	N
*Sox2*	nd	nd	36.79 ± 0.00	16.69	nd	nd	nd
*Syp*	nd	nd	37.24 ± 0.00	17.14	nd	nd	nd
*Tubb3*	39.30 ± 0.00	20.00	36.54 ± 0.00	16.44	3.56	0.08	↓

**Table 6:** Statistical differences identified between groups. Results’ significances are presented through the symbol (*), according to the *p*-value, with one, two, three, or four symbols, corresponding to 0.01 < *p* ≤ 0.05; 0.001 < *p* ≤ 0.01; 0.0001 < *p* ≤ 0.001 and *p* ≤ 0.0001. (ns = no statistically significant differences).

	Neurodifferentiated hDPSCs CM P4	Neurodifferentiated hDPSCs CM P7	CM hDPSCs P4	CM hDPSCs P7	Exosomes hDPSCs P4	Exosomes hDPSCs P7
Neurodifferentiated hDPSCs CM P4		ns	****	**	****	****
Neurodifferentiated hDPSCs CM P7			***	***	****	****
CM hDPSCs P4				****	*	ns
CM hDPSCs P7					****	****
Exosomes hDPSCs P4						ns

## Data Availability

The data that support the findings of this study are available from the corresponding author upon request.

## Abbreviations

The following abbreviations are used in this manuscript:

CM	Conditioned Medium
EDS	Energy Dispersive X-ray spectroscopy
EVs	Extracellular Vesicles
hDPSCs	Human Dental Pulp Stem Cells
hDPSCs-CM	Human Dental Pulp Stem Cells Conditioned Medium
ISEV	International Society for Extracellular Vesicles
MSCs	Mesenchymal Stem Cells
NGC	Nerve Guide Conduit
PNIs	Peripheral Nerve Injuries
RT-PCR	Reverse Transcriptase Polymerase Chain Reaction
SEM	Scanning Electron Microscopy
